# Prion protein (PrP) profiles in blood and CSF: insights into pre-symptomatic and symptomatic prion disease

**DOI:** 10.1093/braincomms/fcag321

**Published:** 2026-08-22

**Authors:** Inês Laginha, Matthias Schmitz, Ângela da Silva Correia, Tayyaba Saleem, Susana da Silva Correia, Saima Zafar, Neelam Younas, Sezgi Canaslan, Stefan Göbel, Elisabeth Root, Maren Breitbarth, Anna-Lisa Fischer, Kathrin Dittmar, Dana Žakova, Peter Hermann, Inga Zerr

**Affiliations:** National Reference Center for CJD Surveillance, Department of Neurology, University Medical Center, 37075 Göttingen, Germany; National Reference Center for CJD Surveillance, Department of Neurology, University Medical Center, 37075 Göttingen, Germany; National Reference Center for CJD Surveillance, Department of Neurology, University Medical Center, 37075 Göttingen, Germany; National Reference Center for CJD Surveillance, Department of Neurology, University Medical Center, 37075 Göttingen, Germany; German Center for Neurodegenerative Diseases (DZNE), Cooperation Unit Translational studies and biomarker, 37075 Göttingen, Germany; National Reference Center for CJD Surveillance, Department of Neurology, University Medical Center, 37075 Göttingen, Germany; National Reference Center for CJD Surveillance, Department of Neurology, University Medical Center, 37075 Göttingen, Germany; National Reference Center for CJD Surveillance, Department of Neurology, University Medical Center, 37075 Göttingen, Germany; National Reference Center for CJD Surveillance, Department of Neurology, University Medical Center, 37075 Göttingen, Germany; National Reference Center for CJD Surveillance, Department of Neurology, University Medical Center, 37075 Göttingen, Germany; National Reference Center for CJD Surveillance, Department of Neurology, University Medical Center, 37075 Göttingen, Germany; National Reference Center for CJD Surveillance, Department of Neurology, University Medical Center, 37075 Göttingen, Germany; National Reference Center for CJD Surveillance, Department of Neurology, University Medical Center, 37075 Göttingen, Germany; National Reference Center for CJD Surveillance, Department of Neurology, University Medical Center, 37075 Göttingen, Germany; National Reference Center for Prion Diseases and Slow Viral Infections, Slovak Medical University, 833 03 Bratislava, Slovakia; National Reference Center for CJD Surveillance, Department of Neurology, University Medical Center, 37075 Göttingen, Germany; National Reference Center for CJD Surveillance, Department of Neurology, University Medical Center, 37075 Göttingen, Germany; German Center for Neurodegenerative Diseases (DZNE), Cooperation Unit Translational studies and biomarker, 37075 Göttingen, Germany

**Keywords:** PrP, plasma, cerebrospinal fluid, fatal familial insomnia, prion disease

## Abstract

The conversion of native prion protein (PrP) into its misfolded isoform, scrapie (PrP^Sc^) and its intracellular accumulation represent central events in the pathogenesis of prion diseases. Reduction of native PrP in the central nervous system (CNS) has emerged as a promising strategy for treatment and prevention of prion diseases in humans. To facilitate translation into clinical practice, it is essential to identify at-risk individuals through biomarker development and to elucidate PrP behaviour across prion disease subtypes and biological fluids. Measurements of PrP in accessible biofluids, such as plasma and cerebrospinal fluid (CSF), may provide a pharmacodynamic readout and enable monitoring for PrP-targeted therapies. This study systematically quantifies PrP in plasma and CSF of individuals with sporadic and genetic prion diseases, healthy controls (HC), patients with non-neurodegenerative neurological conditions (ND) and Alzheimer’s disease (AD). We analysed 136 plasma and 84 CSF samples, including HC, AD, sporadic Creutzfeldt–Jakob disease (sCJD), as well as symptomatic patients and asymptomatic carriers of the mutations D178N, E200K and P102L. Quantification of PrP was performed using a BetaPrion Human ELISA. Statistical analyses assessed differences between diagnostic groups, associations with demographic factors and *PRNP* codon 129 polymorphism, and diagnostic accuracy via ROC curves. Plasma PrP was significantly reduced in patients with sCJD (*P* = 0.043), in symptomatic patients with the E200K (*P* = 0.0078) and in both symptomatic and asymptomatic D178N carriers (*P* = 0.0002) compared to HC. Furthermore, symptomatic and asymptomatic D178N carriers had significantly lower plasma PrP levels than patients with AD (*P* = 0.0025 and *P* = 0.0041, respectively). In CSF, PrP concentrations were notably lower in D178N symptomatic patients (*P* = 0.0026) versus non-neurodegenerative (ND) controls. Plasma PrP levels showed no association with age, sex or disease onset and were lower in genetic prion disease patients with the methionine/valine (MV) genotype at *PRNP* codon 129. The diagnostic accuracy for PrP quantification in plasma as a biomarker discriminated D178N asymptomatic carriers [area under the curve (AUC) = 0.96] and D178N symptomatic patients (AUC = 0.90) from HC with excellent accuracy. In CSF, PrP quantification discriminated D178N symptomatic patients from ND with good accuracy (AUC = 0.64). Taken together, this study defines a characteristic profile of persistently low plasma and CSF PrP in D178N symptomatic and asymptomatic mutation carriers. Low plasma levels in sCJD and E200K, in contrast to P102L, are puzzling. Mutation-specific patterns of PrP need to be considered for monitoring purposes in clinical trials.

## Teaser

Prion protein (PrP) is the key pathogenic protein in prion diseases. Because current therapies aim to lower CNS PrP, understanding PrP dynamics in biofluids across aetiologies is essential. Laginha *et al*. report consistently reduced plasma and CSF PrP in D178N carriers, supporting plasma PrP as a promising pharmacodynamic biomarker for PrP-lowering trials.

## Graphical Abstract

**Graphical Abstract:**
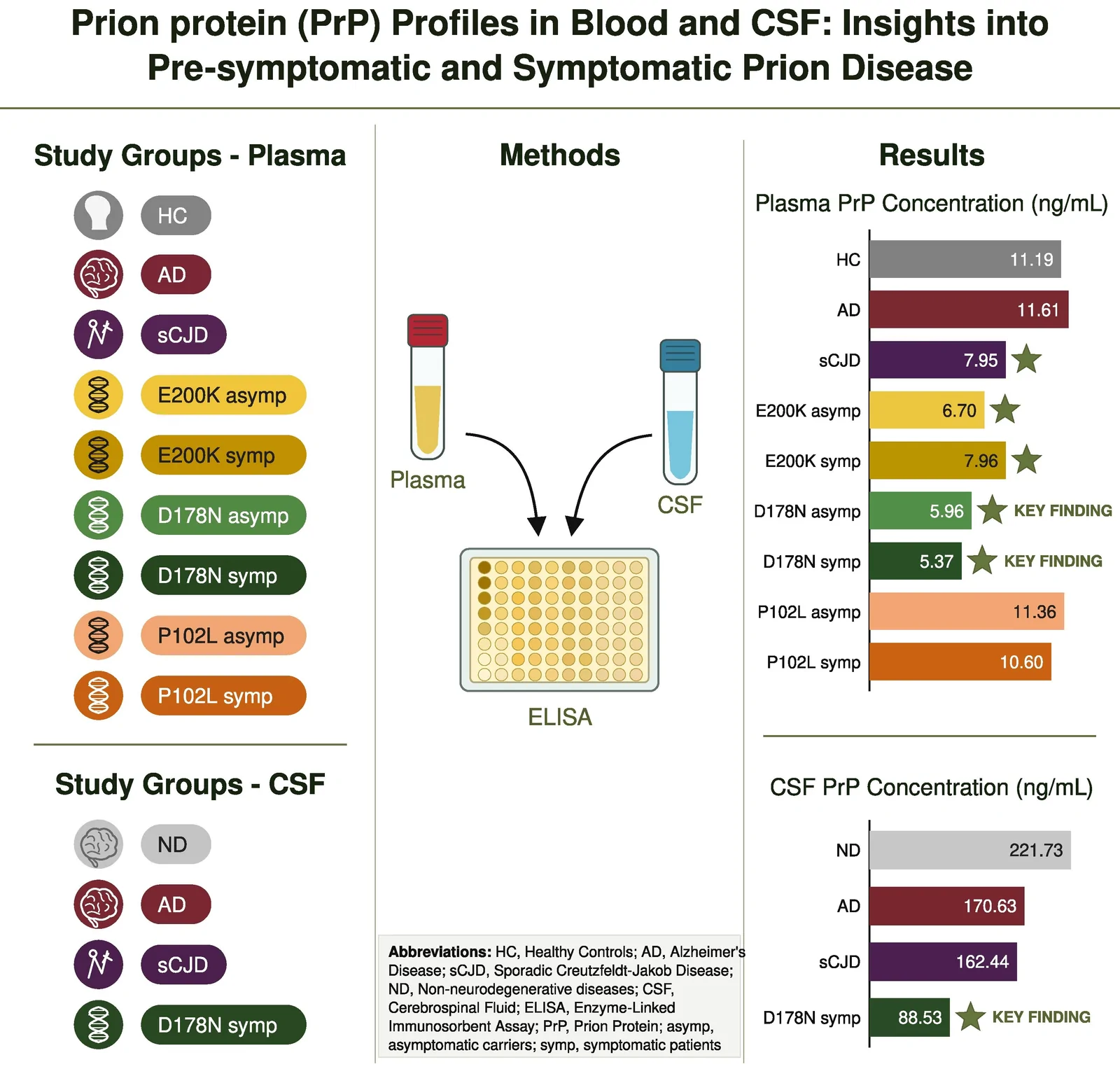

## Introduction

Prion diseases are a group of fatal neurodegenerative disorders that arise in three main aetiological forms: sporadic, genetic and acquired. In all forms, the pathogenic isoform, scrapie prion protein (PrP^Sc^), is generated by misfolding of the normal prion protein (PrP^C^), encoded in the *PRNP* gene and accumulates as aggregates within the central nervous system (CNS).^1^

Sporadic Creutzfeldt–Jakob disease (sCJD) is the most common form, accounting for ∼ 85–90% of human prion disease cases. Inherited prion diseases constitute ∼ 10–15% of cases and are caused by pathogenic variants in the *PRNP* gene, including genetic CJD (gCJD), most frequently linked to the E200K mutation. Gerstmann–Sträussler–Scheinker syndrome (GSS) is typically associated with P102L, and fatal familial insomnia (FFI) is associated with D178N. Acquired forms, such as iatrogenic CJD (iCJD) and variant CJD (vCJD), are rare.^2-4^

The molecular mechanisms that trigger the conversion of PrP^C^ into PrP^Sc^ remain poorly understood. This lack of insight has significant implications, especially for genetic prion diseases, where the transition from a pre-symptomatic to a symptomatic phase cannot currently be predicted. It is hypothesized that once misfolded prion protein (PrP) reaches a critical threshold, it begins to form seeds and aggregates that drive neurodegeneration.

The clinical course of prion disease typically ranges from 5 months to 2 years. Diagnosis is often complicated by the heterogeneity of early symptoms despite a shared underlying disease mechanism. Symptoms can include psychiatric, autonomic, motor and cognitive disturbances, making early recognition particularly challenging.^5^ The diagnosis of prion diseases also relies on cerebrospinal fluid (CSF) biomarkers, including the presence of prion seeding activity through Real-time Quaking-Induced Conversion (RT-QuIC), elevated concentrations of 14-3-3 protein, total tau, neurofilaments (Nfl), as well as alpha-synuclein.^6-11^ Nonetheless, at a pre-symptomatic stage, RT-QuIC signal may be low in people developing FFI and GSS.^12,13^

CSF PrP concentrations are reduced in prion diseases, a characteristic finding across aetiologies, and decline further as the disease progresses, supporting their potential use as biomarkers of disease stage and pharmacodynamic response.^14,15^ Paradoxically, previous studies have reported increased plasma PrP concentrations in prion diseases, although data remain limited and heterogeneous.^16-19^

PrP is known to play a central role in disease pathophysiology. The presence of PrP is essential for the pathogenic transformation into its misfolded isoform, PrP^Sc^.^20^ Only cells that express PrP are susceptible to prion-induced neurotoxicity, and the severity of this toxicity appears to be dose-dependent.^21^ Studies in mouse models have demonstrated that knockout of the PrP gene confers complete resistance to prion disease.^22,23^ Furthermore, reducing PrP expression using gene-editing technologies such as CRISPR or base editors delivered via adeno-associated virus has been shown to extend survival in prion-infected mice.^24^ Conversely, overexpression of PrP in transgenic animals accelerates disease progression.^21^ Several preclinical studies in prion disease models have demonstrated that RNA-targeting strategies, including RNA interference and antisense oligonucleotides (ASOs), can robustly lower PrP expression and delay disease onset or prolong survival.^25-29^ However, the use of ASOs has shown promise in other neurodegenerative diseases characterized by the accumulation and spread of misfolded proteins in a prion-like manner, such as Huntington’s disease and amyotrophic lateral sclerosis (ALS). Intrathecal administration of ASOs in clinical studies has been well-tolerated and led to dose-dependent reductions in mutant huntingtin (HTT) protein in patients with Huntington’s disease, as well as in target transcripts in ALS, without significant adverse effects.^30,31^ These findings have positioned PrP-lowering strategies at the forefront of potential therapeutic approaches.^28,32^ The scientific community increasingly agrees that PrP-lowering therapies represent the most promising path forward. The ASOs in the ongoing trial ‘A Phase 1/2a Study to Evaluate the Safety, Tolerability, Pharmacokinetics and Pharmacodynamics of Intrathecally Administered ION717 in Patients With Prion Disease’, by Ionis Pharmaceuticals, target the mRNA encoding normal PrP, preventing PrP formation.

In prion diseases, as in other neurodegenerative disorders such as Alzheimer’s disease (AD), clinical trial recruitment is shifting from patients with established disease to individuals in asymptomatic or very early symptomatic stages.^33^ Yet the lack of robust biomarkers that precede clinical onset still limits enrolment of individuals who could meaningfully benefit from experimental therapies. Individuals at known high genetic risk, particularly carriers of pathogenic *PRNP* mutations, represent a unique population, allowing longitudinal characterization of biomarker evolution. They also offer a critical window for early therapeutic intervention, when truly preventive or pre-symptomatic treatments may avert irreversible neural damage.

Because lumbar puncture is invasive, CSF is often unavailable for research and trial screening. In this study, we therefore quantified PrP in plasma from individuals with prion disease of diverse aetiologies, spanning pre-symptomatic and symptomatic stages and in CSF from symptomatic patients. As emerging therapeutic strategies increasingly focus on lowering the causal protein PrP, defining PrP dynamics in a blood-based assay becomes critical to understand the disease process, facilitate screening and longitudinal monitoring of treatment effects. Plasma PrP, in particular, may provide a practical pre-symptomatic and pharmacodynamic biomarker for future interventional trials.

## Materials and methods

### Plasma samples

A total of 136 plasma samples were analysed in this study. Blood was collected using EDTA tubes under standardized pre-analytical conditions across all sites. Samples from healthy controls (HC) were obtained at the Department of Transfusion Medicine (*n* = 20) and donated from the Portuguese Association of Prion Diseases (APDP) (*n* = 9).^34^ The healthy control group consisted of voluntary blood donors with no known neurological or other clinically relevant conditions. Samples from patients with AD (*n* = 10) and sCJD (*n* = 21) were collected at the Department of Neurology of the University Medical Center Göttingen (UMG) (Germany) and the National Reference Centre for Transmissible Spongiform Encephalopathies (NRZ TSE).

Blood samples from patients with sCJD were collected for the purpose of diagnosis at the time of diagnosis. They were classified as either probable (*n* = 14) or definite (*n* = 7), based on established diagnostic consensus criteria.^5^

AD was diagnosed according to the criteria defined by the National Institute on Aging-Alzheimer’s Association (NIA-AA) workgroups.^35,36^

Plasma samples from D178N symptomatic patients (*n* = 10), D178N asymptomatic carriers (*n* = 5), P102L symptomatic patients (*n* = 2), P102L asymptomatic carriers (*n* = 3) and E200K symptomatic patients (*n* = 9) were collected for diagnostic purposes in the Department of Neurology of the UMG (Germany) and the NRZ TSE. Blood samples from patients obtained in the UMG were collected in Sarstedt EDTA tubes and stored in 2 ml Sarstedt SafeSeal PP microtubes.

The E200K symptomatic patient group also contained samples obtained from the Department of Prion Diseases, Slovak Medical University (SZU), Bratislava, Slovakia (*n* = 27). Plasma samples from an E200K asymptomatic carrier were obtained from APDP (*n* = 1) and from the National Reference Center for prion diseases and slow viral infections, SZU, Bratislava, Slovakia (*n* = 2). Blood samples from symptomatic patients and asymptomatic E200K mutation carriers were collected in different hospitals and in various types of tubes, most frequently in sterile PS tubes. They were aliquoted and stored in 2 ml Sarstedt SafeSeal PP microtubes. Each sample was frozen and thawed approximately twice. Samples from asymptomatic mutation carriers were stored for 9 years, and from symptomatic patients for 1–4 years.

Plasma samples from D178N asymptomatic carriers (*n* = 8) were donated by APDP. These blood samples were collected in Sarstedt EDTA-tubes and stored in 2 ml Sarstedt SafeSeal PP microtubes.

Only up to two samples were available for a few individuals, and sampling time points were not systematically recorded; consequently, a true longitudinal analysis was not possible (Supplementary Table 1). For patients with two available samples, the mean PrP concentration was used.

For clarity and consistency, participants carrying a pathogenic prion-disease–associated mutation will be designated by their specific mutation and clinical status (asymptomatic carrier or symptomatic patient).

For each participant, a unique randomization number was assigned.

### CSF samples

CSF samples from 84 patients were used to measure PrP. The CSF samples from patients with primarily non-neurodegenerative neurological diseases (ND *n* = 25), AD (*n* = 11), sCJD (*n* = 20), probable (*n* = 13) or definite (*n* = 7), D178N symptomatic patients (*n* = 10), as well as a single D178N asymptomatic carrier (*n* = 1) were obtained at the NRZ TSE for diagnostic purposes.^5^

The ND group comprised patients with neurological conditions not associated with neurodegenerative pathology, such as chronic pain syndrome, Miller–Fisher syndrome, cranial nerve palsy, stroke, exclusion of neuroborreliosis, benign paroxysmal positional vertigo, exclusion of viral meningitis, familial hemiplegic migraine, lumbar herniation, polyneuropathy, spinal haematoma, idiopathic intracranial hypertension (Supplementary Table 1).

### Plasma and CSF PrP quantification

PrP in plasma and CSF samples was measured using a commercially available enzyme-linked immunosorbent assay (ELISA) specific for human PrP—BetaPrion Human ELISA (Roboscreen), according to the manufacturer’s instructions. Plasma samples were diluted 1:4, and CSF samples were diluted 1:20.

### Genetic tests

Genetic prion diseases were diagnosed according to established surveillance criteria, following prion protein gene (*PRNP*) analysis and World Health Organization (WHO) criteria.^37^

### Statistical analysis

For statistical analyses, GraphPad Prism 10 for macOS was used. For distribution testing, D'Agostino–Pearson and Shapiro-Wilk tests were used. Data were analysed using Kruskal–Wallis, followed by Dunn’s post hoc test for multiple comparisons. For two-group comparisons of biomarker levels, non-parametric Mann–Whitney U tests were used. Spearman rank correlation coefficients were used to assess associations between continuous biomarker levels. For the analysis of differences in PrP concentrations between different cohorts, only cohorts including enough cases to perform normality tests were used. In order to estimate the diagnostic accuracy of PrP, receiver operating characteristic (ROC) curve analyses were performed, and area under the curve (AUC) with 95% confidence intervals (95% CI) were calculated. ROC curve comparisons were performed.

### Ethics approval and consent to participate

The study was conducted according to the revised Declaration of Helsinki and Good Clinical sPractice guidelines and approved by all Ethics committees (Reference numbers 11/11/93, 5/09/08, 9/06/08, 19/11/09 University Medical Center Göttingen, Germany). All study participants or their legal guardians provided written informed consent. This manuscript was developed according to the STROBE checklist for reports of cohort studies.

## Results

### Characterization of the PrP ELISA assay

PrP in plasma and CSF samples was measured using BetaPrion Human ELISA (Roboscreen) as described in the Materials and methods section.

The range of measurement in tested plasma samples was 2.774 (minimum) and 19.39 (maximum) ng/ml. The range of measurement in tested CSF samples was 12.90 (minimum) and 401.4 (maximum) ng/ml.

### Plasma PrP levels

Plasma PrP was measured in HC, AD, sCJD, in symptomatic and asymptomatic cases of the three most prevalent forms of genetic prion diseases (E200K, D178N and P102L mutations).

**Figure 1:**
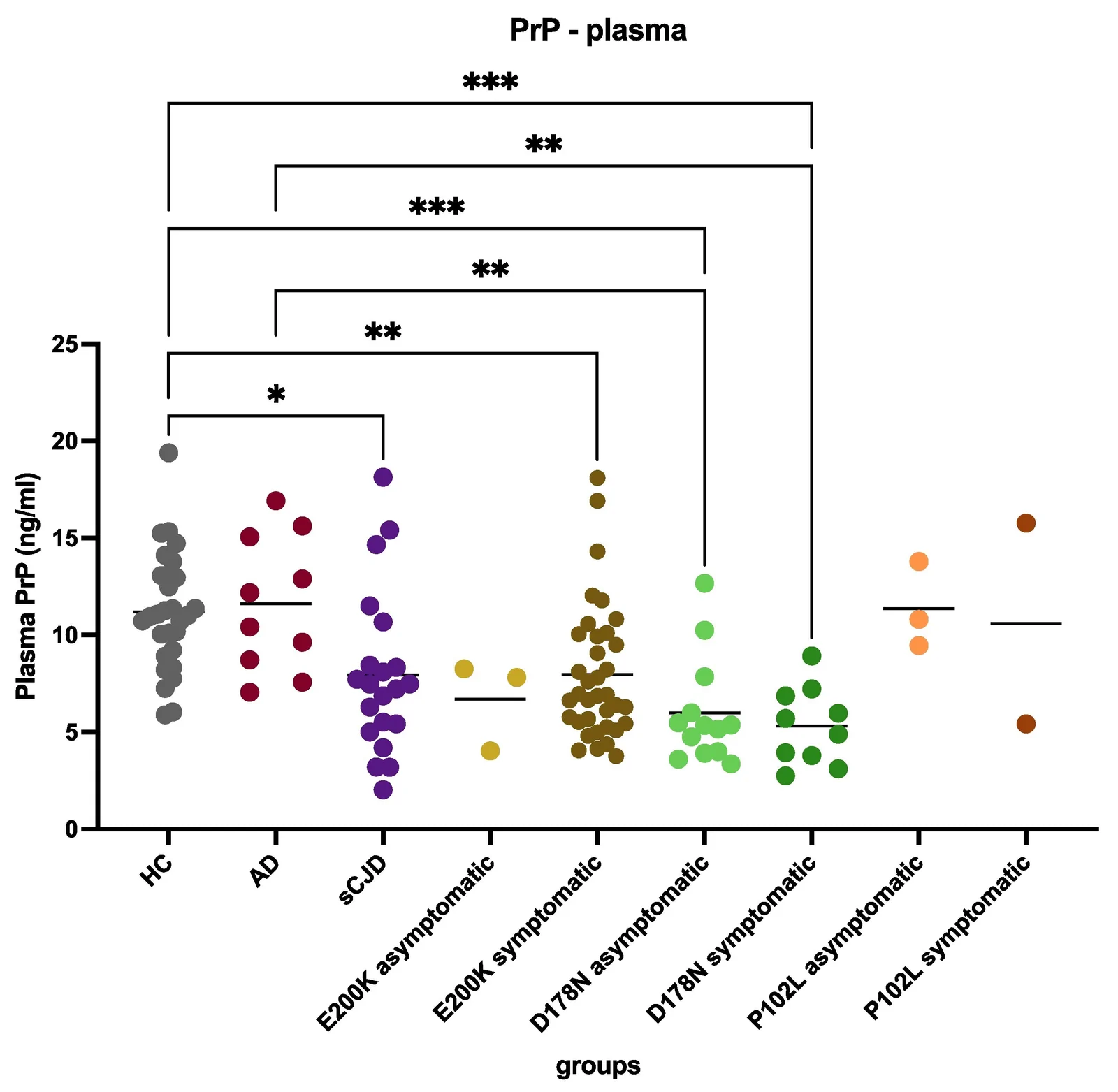
**Plasma prion protein (PrP) concentrations in healthy controls (HC) (*n* = 29), patients with Alzheimer’s disease (AD) (*n* = 10), patients with sporadic Creutzfeldt–Jakob disease (sCJD) (*n* = 21) as well as individuals harbouring classic prion disease mutations both in a symptomatic and asymptomatic phase—D178N (symptomatic *n* = 10; asymptomatic *n* = 13), P102L (symptomatic *n* = 2; asymptomatic *n* = 3) and E200K (symptomatic *n* = 36; asymptomatic *n* = 3).** Each datapoint represents an independent measure from a single patient. Compared to HC, plasma PrP concentrations were lower in sCJD (*P* = 0.0403), in E200K symptomatic patients (*P* = 0.0078) and in D178N mutations, both symptomatic and asymptomatic (*P* = 0.0002). Plasma PrP levels were also lower in D178N symptomatic patients (*P* = 0.0025) and in D178N asymptomatic carriers (*P* = 0.0041) compared to AD patients. Kruskal–Wallis test and Dunn’s post hoc test (correction for multiple testing) were applied. *P < 0.05, **P < 0.01, ***P < 0.001.

In order to assess whether there were differences in plasma PrP levels among diagnostic groups, PrP concentrations were analysed in a multiple comparison test. Kruskal–Wallis testing indicated an overall difference in PrP concentrations across the nine groups (*P* < 0.0001). Compared to HC, plasma PrP concentrations were lower in sCJD (*P* = 0.0403), in E200K symptomatic patients (*P* = 0.0078) and in D178N mutation carriers, both symptomatic patients and asymptomatic carriers (*P* = 0.0002). No significant difference was found between HC and P102L mutation cases, both symptomatic (*n* = 2; *P* > 0.99) and asymptomatic (*n* = 3; *P* = 0.27). PrP levels were also lower in D178N symptomatic patients (*P* = 0.0025) and in D178N asymptomatic carriers (*P* = 0.0041) compared to AD patients. No further statistical differences were observed between AD patients and other groups (Fig. 1).

Lowest PrP concentrations were found in D178N symptomatic patients (5.37 ± 2.01 ng/ml) and D178N asymptomatic carriers (5.96 ± 2.76 ng/ml), but no statistical differences among different forms of prion diseases were detected. The highest PrP concentrations were found in HC (11.19 ± 3.01) and in AD (11.61 ± 3.48) (Table 1).

**Table 1:** Demographic plasma PrP data from the study population

	Number of cases (*n*)	Gender (f/m)	Age	PrP (ng/ml) mean ± SD	PrP (ng/ml) 95% CI
HC	29	14/15	52 ± 10	11.19 ± 3.01	10.05, 12.34
sCJD	21	7/14	66 ± 10	7.95 ± 4.16	6.05, 9.84
AD	10	6/4	67 ± 9	11.61 ± 3.48	9.12, 14.10
E200K asymptomatic	3	1/2	40 ± 4	6.70 ± 2.32	0.94, 12.46
E200K symptomatic	36	24/12	62 ± 7	7.96 ± 3.46	6.79, 9.13
P102L asymptomatic	3	0/3	48 ± 17	11.36 ± 2.22	5.85, 16.86
P102L symptomatic	2	1/1	56 ± 16	10.60 ± 7.32	−55.14, 76.34
D178N asymptomatic	13	9/4	47 ± 12	5.96 ± 2.76	4.29, 7.63
D178N symptomatic	10	2/8	52 ± 13	5.37 ± 2.01	3.93, 6.80

Number of cases (*n*), age in years [mean values ± standard deviation (SD), sex (female (f)/male (m)], plasma PrP concentrations [mean ± standard deviation (SD) in ng/ml] and 95% CI (in ng/ml) are indicated. Mutations in the PRNP gene are indicated for genetic prion diseases (gPD). Healthy controls (HC), sporadic Creutzfeldt–Jakob disease (sCJD), Alzheimer’s disease (AD).

**Figure 2:**
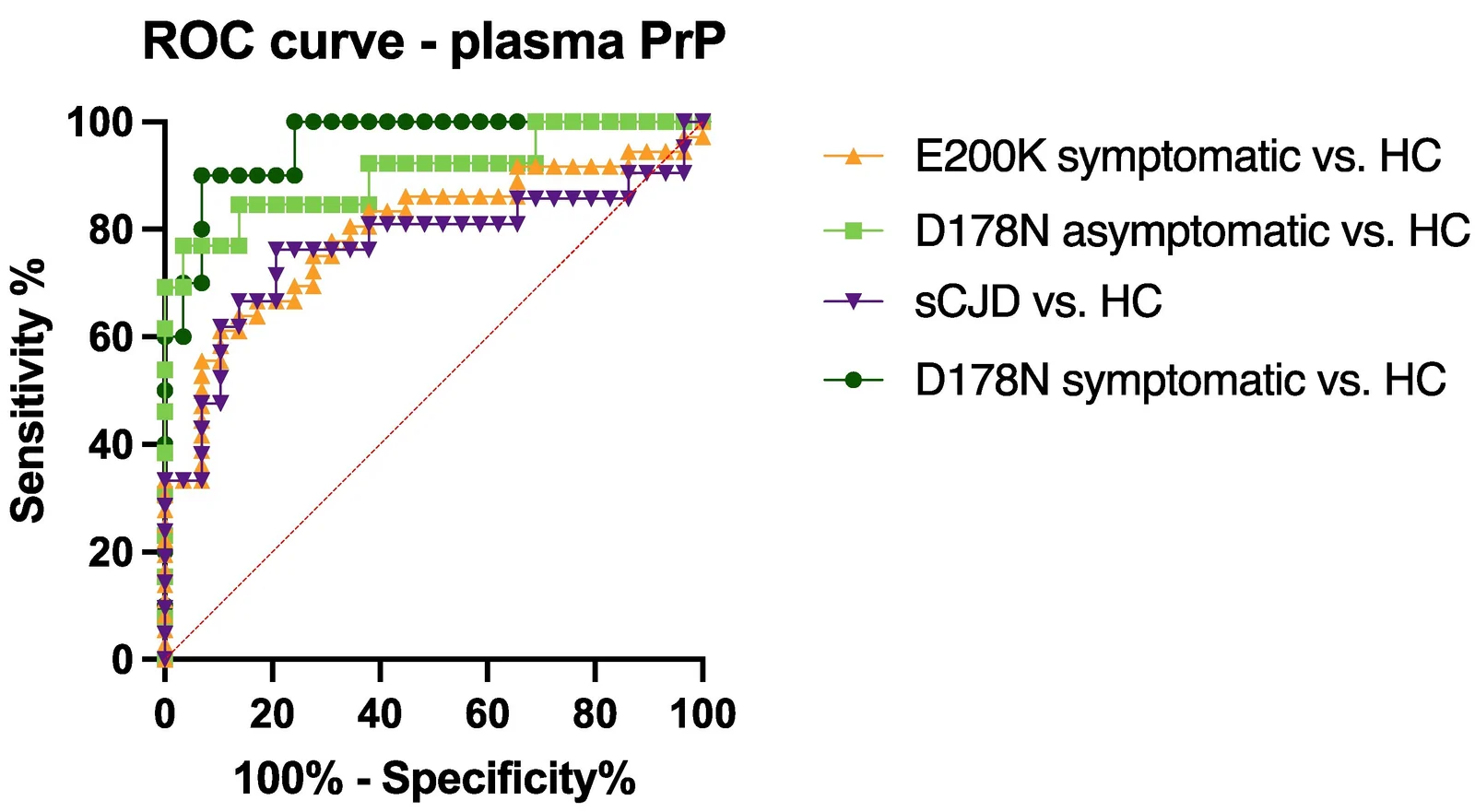
**Receiver operating characteristic (ROC) curves for plasma prion protein (PrP) concentrations in E200K symptomatic patients (*n* = 36), D178N asymptomatic carriers (*n* = 13), sporadic Creutzfeldt–Jakob disease (sCJD) (*n* = 21) and D178N symptomatic patients (*n* = 10) versus the healthy controls (HC) group (*n* = 29).** D178N symptomatic patients and D178N asymptomatic carriers were discriminated from HC with excellent accuracy (D178N symptomatic patients: AUC = 0.96, 95% CI: 0.90–1.00 and D178N asymptomatic carriers: AUC = 0.90, 95% CI: 0.79–1.00). E200K symptomatic patients and sCJD patients were discriminated from HC with good accuracy (E200K symptomatic: AUC = 0.79, 95% CI: 0.68–0.90 and sCJD: AUC = 0.77, 95% CI: 0.62–0.92). Area under the curve (AUC) and 95% confidence interval (CI) are shown for PrP analysis.

The diagnostic accuracy of plasma PrP quantification as a biomarker for prion diseases was determined from AUC values derived from the comparison of each pair of diagnostic groups. D178N symptomatic patients and D178N asymptomatic carriers were discriminated from HC with excellent accuracy (D178N symptomatic patients: AUC = 0.96, 95% CI: 0.90–1.00 and D178N asymptomatic carriers: AUC = 0.90, 95% CI: 0.79–1.00). E200K symptomatic patients and sCJD patients were discriminated from HC with good accuracy (E200K symptomatic: AUC = 0.79, 95% CI: 0.68–0.90 and sCJD: AUC = 0.77, 95% CI: 0.62–0.92) (Fig. 2).

For all symptomatic D178N carriers (*n* = 10), paired plasma and CSF samples were available. Spearman’s rank correlation showed no significant association between plasma and CSF PrP concentrations (*r*s = 0.23, *P* = 0.26, one-tailed; data not shown), providing no evidence of a monotonic relationship between compartments. In the sCJD cohort, 5 of 21 samples originated from a single patient. Spearman correlation assessing the correspondence between compartments was not significant (*r*s = −0.2, *P* = 0.39, one-tailed), indicating no proportional relationship in PrP concentrations (data not shown). In both cohorts, the date of biofluid collection was not taken into consideration.

### Correlations of demographic, genetic, collection date and different cohorts with PrP concentrations

**Figure 3:**
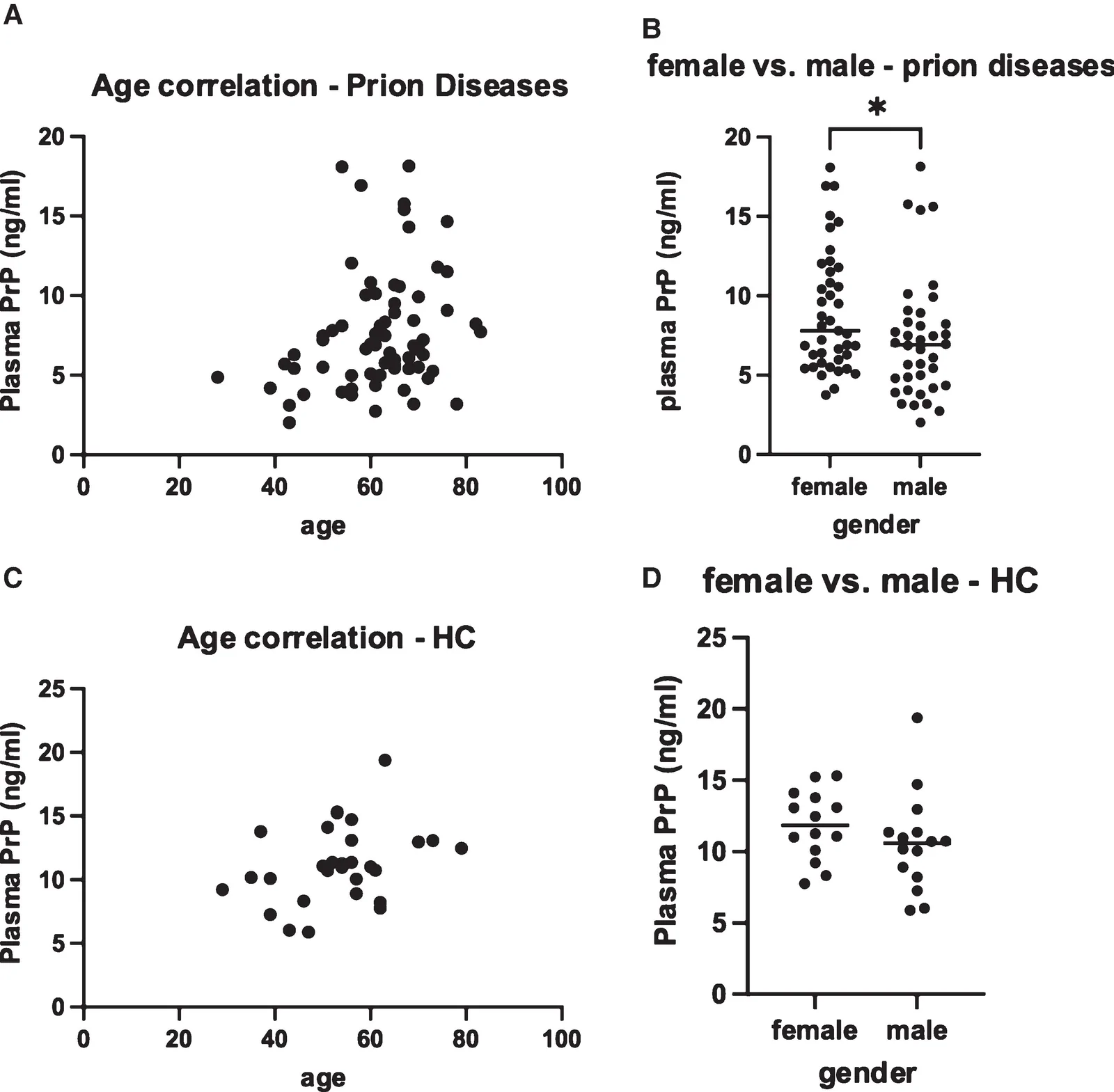
**Association between age of disease onset and gender on plasma prion protein (PrP).** Each datapoint represents an independent measure from a single patient. (**A**) Relationship analysis between PrP levels and age at disease onset in prion disease (*n* = 69): sporadic Creutzfeldt–Jakob disease (sCJD) (*n* = 21), D178N symptomatic patients (*n* = 10), P102L symptomatic patients (*n* = 2) and E200K symptomatic patients (*n* = 36). Plasma PrP levels showed a correlation with age at onset (*P* = 0.0255, r = 0.2689). Spearman rank correlation test was used. (**B**) PrP in prion disease (sCJD) (*n* = 21), D178N symptomatic patients (*n* = 10), P102L symptomatic patients (*n* = 2) and E200K symptomatic patients (*n* = 36) stratified by gender. Female patients displayed significantly higher PrP concentrations than the male counterparts (*P* = 0.0329). Mann–Whitney U test was used. (**C**) The healthy control (HC) group (*n* = 29) lacked an association between plasma PrP levels and age at blood collection (*P* = 0.1273, *r* = 0.2898). Spearman rank correlation test was used. (**D**) The HC control group (*n* = 29) also lacked an association between plasma PrP levels and gender (*P* = 0.1337). Mann–Whitney U test was used.

In symptomatic prion disease patients, plasma PrP levels correlated with age at onset (*P* = 0.0255; Fig. 3A). Female patients had significantly higher plasma PrP concentrations than males (*P* = 0.0329; Fig. 3B). When healthy mutation carriers were included, this sex difference was no longer significant (*P* = 0.1063; data not shown). The HC control group also lacked association with age (*P* = 0.1273) (Fig. 3C) and sex (*P* = 0.1337) (Fig. 3D).

In order to take preanalytic disparities into account, whenever samples were obtained from different places, plasma PrP concentrations were compared. We analysed plasma PrP concentrations in a subset of the D178N asymptomatic carriers (UMG versus APDP cohorts), E200K symptomatic patients (UMG versus SZU cohorts) and HCs (APDP versus UMG, Department of Transfusion Medicine). In D178N asymptomatic carriers, PrP levels from UMG cases were similar to those from the APDP cohort (*P* = 0.8329). Nevertheless, E200K symptomatic patient samples showed significant differences (*P* = 0.0098). Also, HC samples showed significant differences (*P* = 0.018) regarding PrP concentrations (APDP versus UMG). With the exception of the HC cohorts, where individuals from the APDP cohort were significantly younger than the UMG cohort (*P* = 0.0004), the ages of individuals in the two cohorts of D178N asymptomatic carriers and the E200K symptomatic patients showed no significant differences (Supplementary Fig. 1). While the differences in concentrations among HC in the two cohorts can be attributed to age differences, the variations observed among E200K symptomatic patients are most likely due to differences in sample handling procedures, such as the number of freeze–thaw cycles and tube transfers.

No statistical differences in plasma PrP levels were found between E200K symptomatic patients and E200K asymptomatic carriers (*P* > 0.9999). Due to the low number of asymptomatic E200K mutation carriers (*n* = 3), it is difficult to draw any meaningful conclusion. In the P102L mutation, PrP concentrations were similar between asymptomatic carriers (*n* = 3) and symptomatic patients (*n* = 2), in agreement with the absence of alterations between ND and symptomatic cases described above.

**Figure 4:**
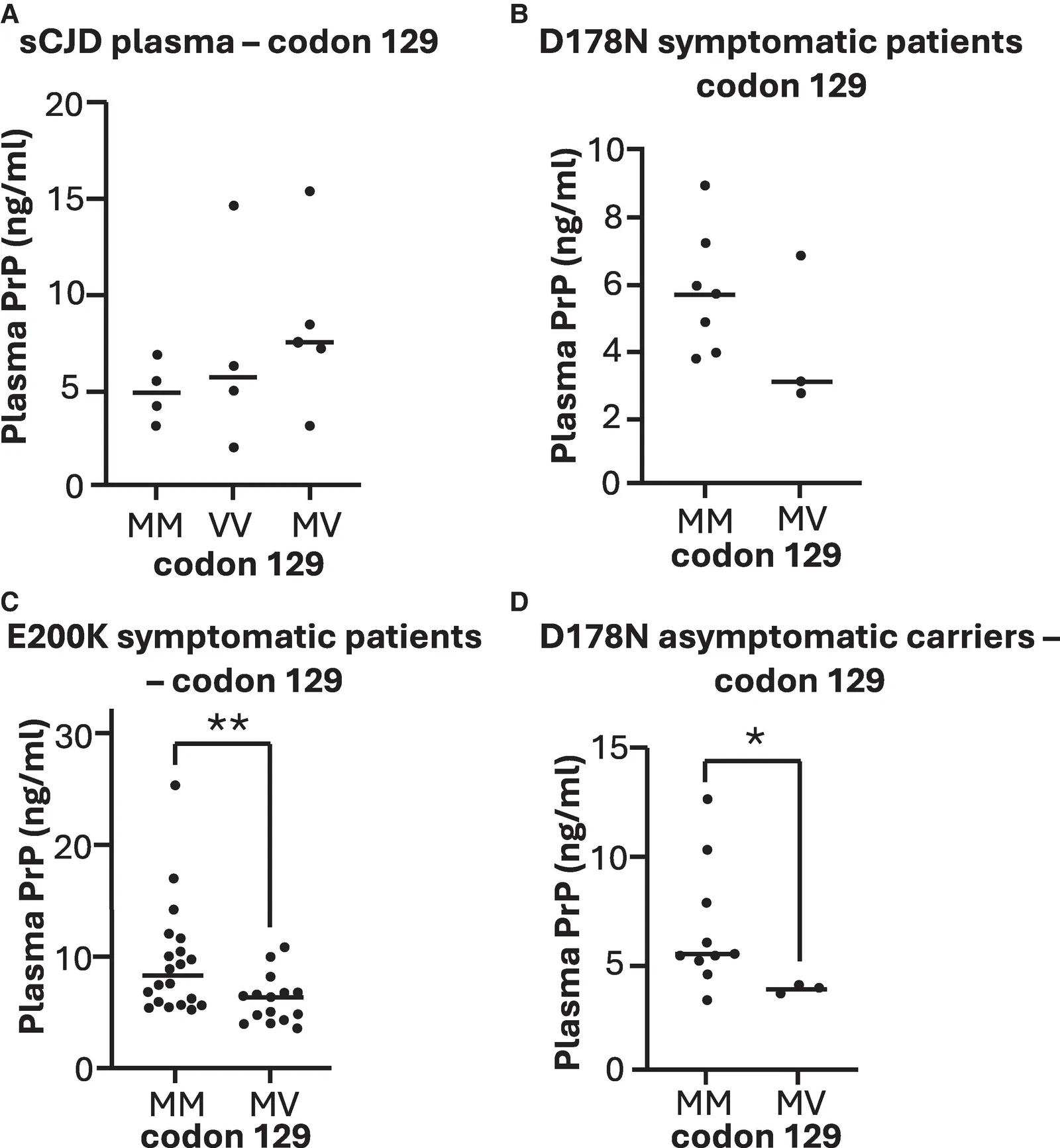
**Plasma prion protein (PrP) concentrations according to codon 129 polymorphism (M methionine, V valine).** Each datapoint represents an independent measure from a single patient. (**A**) Among the 21 patients with sporadic Creutzfeldt–Jakob disease (sCJD), the codon 129 genotype was known for 13 patients (MM *n* = 4; VV *n* = 4; MV *n* = 5). No significant differences were observed regarding plasma PrP levels in different codon 129 genotypes (*P* = 0.26). (**B**) The information about codon 129 genotype was available for all D178N symptomatic patients (*n* = 10; MM *n* = 3; MV *n* = 7) showed no significant differences regarding plasma PrP levels in different codon 129 genotypes (*P* = 0.27). (**C**) From 36 E200K symptomatic patients, the information about codon 129 genotype was known for 35. Significantly lower plasma PrP concentrations were observed in patients with MV at position 129 of *PRNP*, compared to those with MM (MV 6.25 ± 2.15 ng/ml, *n* = 15; MM 9.60 ± 4.93 ng/ml, *n* = 20; *P* = 0.009), (**D**) The codon 129 genotype was known for all 13 D178N asymptomatic carriers. Significantly lower plasma PrP concentrations were observed in patients with MV at position 129 of *PRNP*, compared to those with MM (MV 3.83 ± 0.20 ng/ml, *n* = 3; MM 6.60 ± 2.86 ng/ml, *n* = 10; *P* = 0.049). Kruskal–Wallis test followed by Dunn’s post hoc test for multiple comparisons and Mann–Whitney U test for two-group comparisons.

In order to evaluate whether *PRNP* codon 129 genotype displayed any influence on plasma PrP levels, sCJD, D178N symptomatic patients, E200K symptomatic patients and D178N asymptomatic carriers were stratified in methionine/methionine (MM), methionine/valine (MV) and valine/valine (VV) genotypes. In the sCJD and D178N symptomatic patient cohorts we observed no significant differences regarding plasma PrP levels in different codon 129 genotypes (sCJD: *P* = 0.26; D178N symptomatic patients: *P* = 0.27). The E200K symptomatic patient and the D178N asymptomatic carrier cohort displayed significantly lower plasma PrP concentrations in patients with MV at position 129 of *PRNP*, compared to those with MM (E200K symptomatic patients: MV 6.25 ± 2.15 ng/ml, *n* = 15; MM 9.60 ± 4.93 ng/ml, *n* = 20; *P* = 0.0095; D178N asymptomatic carriers: MV 3.83 ± 0.20 ng/ml, *n* = 3; MM 6.60 ± 2.86 ng/ml, *n* = 10; *P* = 0.049) (Fig. 4).

### CSF PrP levels

**Figure 5:**
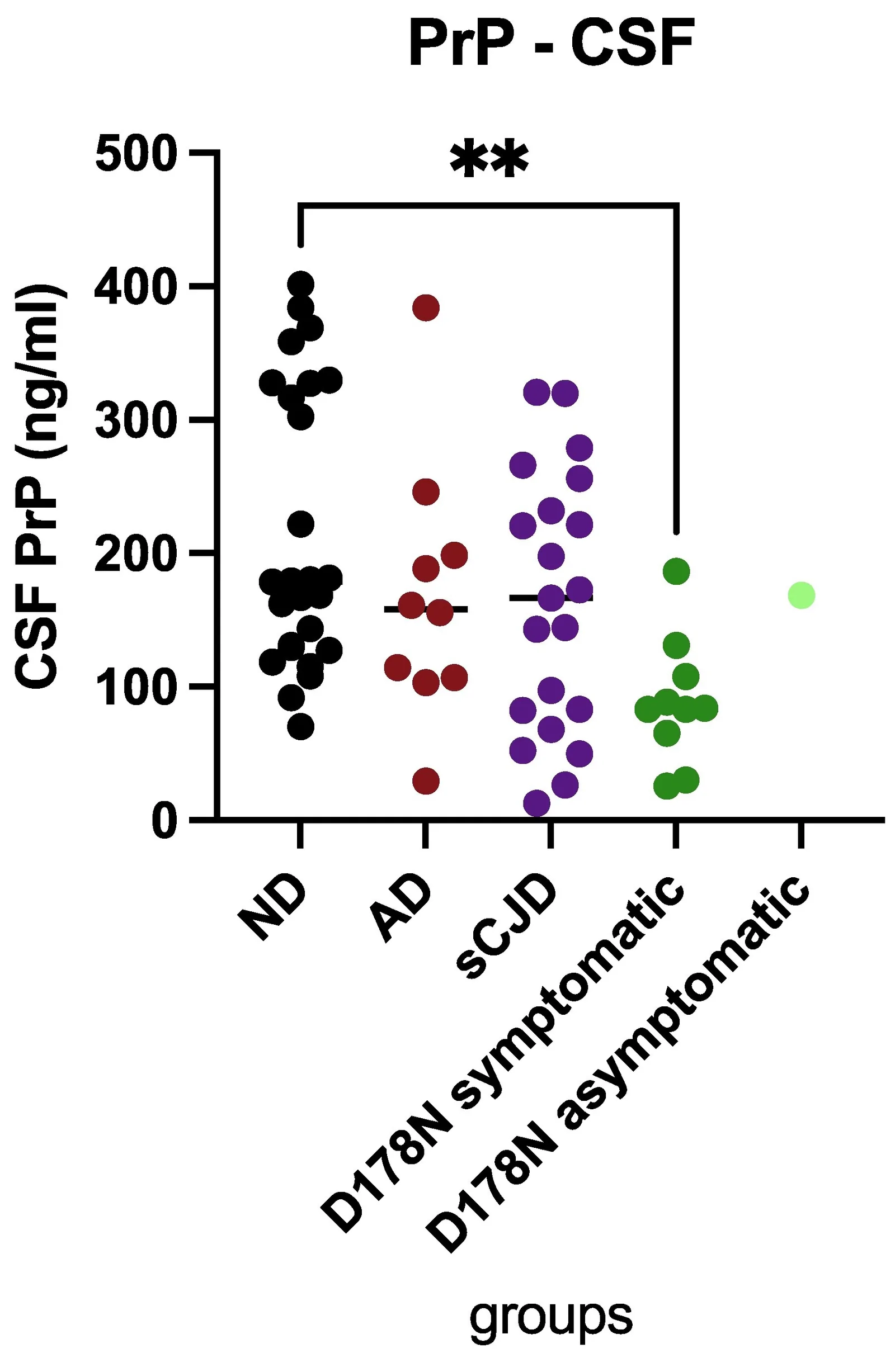
**CSF prion protein (PrP) concentrations in patients with non-neurodegenerative neurological conditions (ND) (*n* = 26), Alzheimer’s disease (AD) (*n* = 10), sporadic Creutzfeldt–Jakob disease (sCJD) (*n* = 21), D178N symptomatic patients (*n* = 10) and a single D178N asymptomatic carrier.** Each datapoint represents an independent measure from a single patient. Compared to ND, CSF PrP concentrations were lower in D178N symptomatic patients (*P* = 0.0045). Kruskal–Wallis test and Dunn’s post hoc test (correction for multiple testing) were applied. **P < 0.01.

CSF PrP concentrations were measured in ND, AD, sCJD, D178N symptomatic patients and D178N asymptomatic carriers. PrP levels were lower in AD, sCJD and D178N symptomatic patients compared to non-neurodegenerative controls, being lowest in D178N symptomatic patients. In order to assess whether there were differences in PrP levels among diagnostic groups, ND, AD, sCJD and D178N symptomatic patient groups were further analysed in a multiple-comparison test. Kruskal–Wallis testing indicated an overall difference in PrP concentrations across the five groups (*P* = 0.0134). Compared to ND, PrP was lower in D178N symptomatic patients (*P* = 0.0045). No further statistical differences were detected (Fig. 5; Table 2).

**Table 2:** Demographic and CSF PrP data from the study population

	Number of cases (*n*)	Gender (f/m)	Age (mean ± SD)	PrP (ng/ml) mean ± SD	PrP (ng/ml) 95% CI
Non-neurodegenerative diseases (ND)	26	16/10	57.54 ± 16.00	221.73 ± 103.67	181.53, 261.93
AD	11	8/3	74 ± 6.82	170.53 ± 93.00	108.06, 233.01
sCJD	20	13/7	69 ± 8.74	162.44 ± 96.97	118.30, 206.58
D178N symptomatic patients	10	2/8	53 ± 13	88.53 ± 46.92	54.97, 122.1
D178N asymptomatic carriers	1	1/0	30	168.44	

Number of cases (*n*), age in years [mean values ± standard deviation (SD), sex (female (f)/male (m)], CSF PrP concentrations [mean ± standard deviation (SD) in ng/ml] and 95% CI (in ng/ml) are indicated. Mutations in the PRNP gene are indicated for genetic prion diseases (gPD). Non-neurodegenerative diseases (ND), sporadic Creutzfeldt–Jakob disease (sCJD), Alzheimer’s disease (AD).

**Figure 6:**
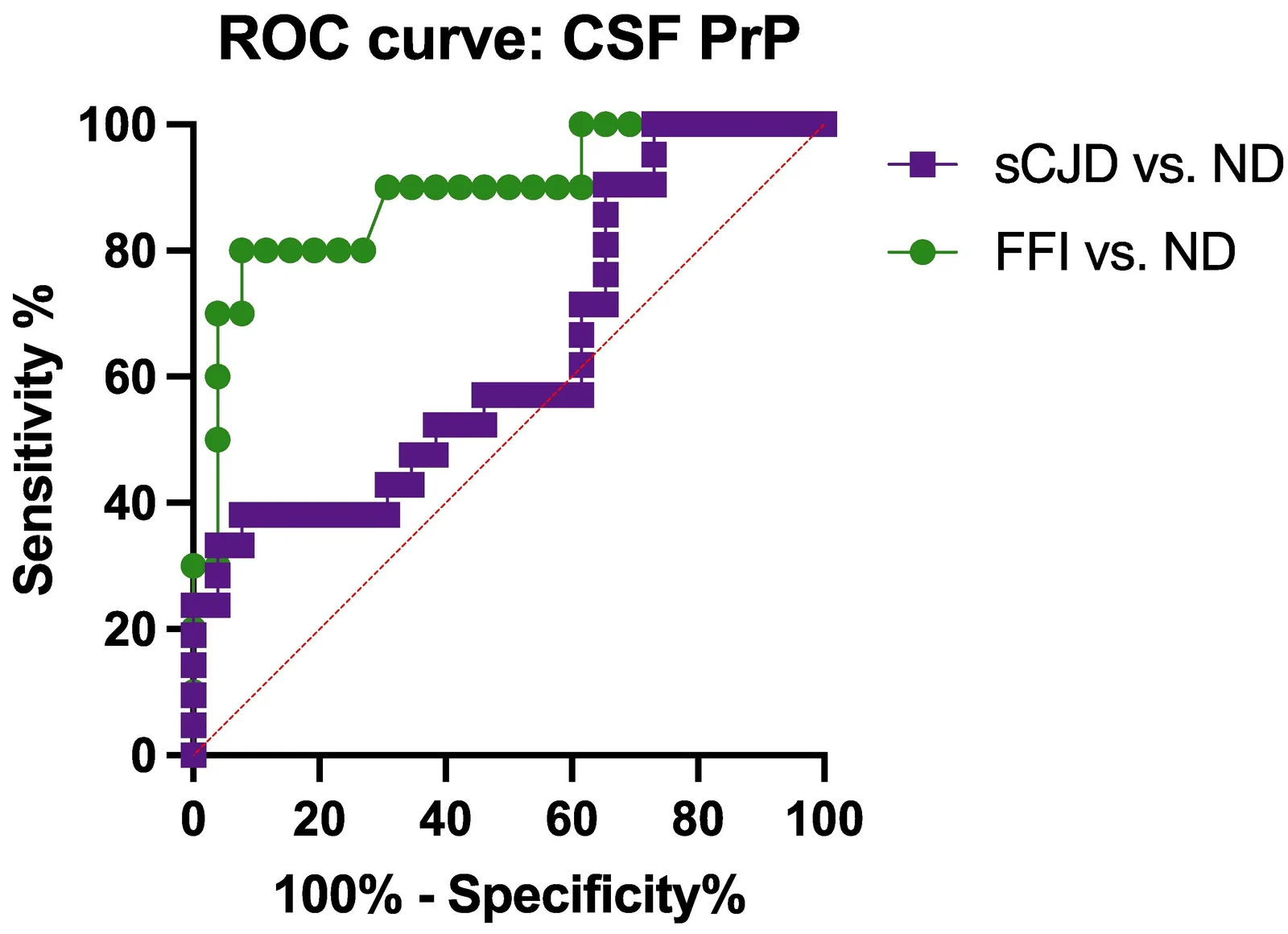
**Receiver operating characteristic (ROC) curves for CSF prion protein (PrP) concentrations in sporadic Creutzfeldt–Jakob disease (sCJD) (*n* = 21) and D178N symptomatic patients (*n* = 10) versus the non-neurodegenerative neurological conditions (ND) group) (*n* = 26).** D178N symptomatic patients were discriminated from ND with good accuracy (AUC = 0.89, 95% CI: 0.76–1.00). sCJD cases showed a poor discriminatory accuracy compared to ND (AUC = 0.64, 95% CI: 0.90–1.00). Area under the curve (AUC) and 95% confidence interval (CI) are shown for PrP analysis.

Regarding the diagnostic accuracy of CSF PrP as a biomarker, D178N symptomatic patients were discriminated from ND with good accuracy (AUC = 0.89, 95% CI: 0.76–1.00). However, sCJD cases showed a poor discriminatory accuracy compared to ND (AUC = 0.64, 95% CI: 0.90–1.00) (Fig. 6).

To assess whether the *PRNP* codon 129 genotype had any impact on CSF PrP levels, we stratified sCJD and D178N symptomatic patients according to genotype and were not able to observe any differences (sCJD: *P* = 0.26; D178N symptomatic patients: *P* = 0.25) (Supplementary Fig. 2).

## Discussion

PrP is expressed in both peripheral tissues and the CNS. Its concentrations in blood and CSF are responsive to CNS injury, including ischaemic stroke,^38^ subarachnoid haemorrhage, and traumatic brain injury,^39^ correlating with both severity and poor clinical outcomes. Experimental evidence shows that PrP is enzymatically stable and capable of crossing the blood–brain barrier (BBB) bidirectionally via a saturable transport mechanism.^40^ This bidirectional transport facilitates exchange between the peripheral circulation and the CNS, likely contributing to PrP’s physiological functions and its role in prion disease pathogenesis. Although PrP concentrations are higher in CSF than in plasma, such ongoing exchange across the BBB suggests that PrP levels tend to show parallel trends in both CSF and plasma, reflecting systemic rather than isolated compartment changes.^37^

Quantification of PrP in various biological fluids becomes important in view of emerging PrP-lowering therapies in the prion field. Specifically, detailed characterization of PrP profiles across preclinical and clinical stages of prion diseases is needed, particularly in easily accessible biofluids such as plasma. Wherever paired plasma and CSF samples were available from the same patient, we performed a correlation analysis. In symptomatic D178N carriers, all patients (*n* = 10) had both plasma and CSF available. We observed no significant correlation between PrP concentrations in the two compartments, which may reflect independent regulation of PrP in CSF and plasma. An alternative explanation is that PrP varies substantially over time; if plasma is to track CNS changes, samples will need to be collected at closely matched time points, underscoring the value of longitudinal designs.

For diagnostic purposes, detection of PrP^Sc^ seeding activity in CSF using RT-QuIC has become a highly specific and sensitive diagnostic tool.^41^ Additional supportive CSF biomarkers include elevated levels of 14-3-3 protein and total tau, which reflect neuronal damage, although they lack disease specificity.^5,11,42^ Given the invasiveness and limitations of CSF-based tests, there has been growing interest in developing blood-based biomarkers. Although the metabolites associated with neurodegeneration are present in lower concentrations in peripheral blood than in CSF, blood analyses are more accessible and amenable to repeated sampling, making them particularly valuable for early detection and longitudinal monitoring.

Here, we characterize PrP profiles with an ELISA-based assay, focusing our assessment on symptomatic and pre-symptomatic individuals carrying various pathogenic *PRNP* mutations.

Plasma PrP levels were significantly reduced in symptomatic prion disease patients in whom the cohort size was adequate to permit robust statistical analysis. Also, in D178N asymptomatic carriers, plasma PrP concentrations were significantly lower than in HC. They were comparable to symptomatic D178N patients. PrP concentrations were not significantly different between HC and AD patients.

Similar to the findings for plasma PrP levels, CSF from ND cases showed significantly higher PrP concentrations compared with D178N symptomatic patient cases, but not in comparison with AD or sCJD. Except for one D178N asymptomatic carrier—who underwent lumbar puncture (LP) due to suspected disease onset that was later ruled out—CSF samples from asymptomatic *PRNP* mutation carriers were not available, as LPs are not routinely performed in this population.

In our study, we demonstrated that plasma PrP differentiated D178N symptomatic patients and D178N asymptomatic carriers from HC with excellent diagnostic accuracy, and E200K symptomatic patients as well as sCJD patients with moderate diagnostic accuracy. CSF PrP is able to discriminate FFI patients from ND with good diagnostic accuracy, but not sCJD patients.

A matched-pairs analysis of PrP concentrations in plasma and CSF from all symptomatic D178N patients (*n* = 10) revealed that concentrations are not proportional between compartments. These findings suggest that plasma levels do not reflect PrP concentration changes in CSF. Nevertheless, we cannot exclude the possibility that CSF and plasma samples were not obtained at the same timepoint.

Llorens *et al*.^17^ and Villar-Piqué *et al*.^14^, respectively, described cases where plasma PrP was elevated while CSF PrP concentrations were reduced in D178N symptomatic patients.^14,17^ Unlike those reports, our study did not reveal a dissociation between plasma and CSF PrP levels, but a consistent pattern of lower PrP levels in both plasma and CSF among D178N symptomatic patients.^17^ This is in line with previous studies on a smaller number of patients.^14,18,19^ Whether this alteration reflects a stable constitutional trait or represents part of a continuum that evolves alongside disease progression remains unresolved. Others have attributed this observation to a preferential degradation of the D178N mutant PrP independent of disease progression.^43,44^ Another option to discuss is the idea that the Betaprion ELISA kit (which uses 3F3 as the capture antibody and 15F5 as the detection antibody)^45^ might have reduced sensitivity for the D178N variant relative to other PrP forms because its epitope has been mapped to a region adjacent to D178N.^18,46^ In this context, the epitope may be occluded due to protein misfolding or increased proteolytic cleavage associated with disease. Although the assay is described as measuring total PrP, we cannot exclude that its sensitivity is reduced for certain conformations and proteolytic fragments or isoforms of truncated species relevant in the disease.^47-49^ Importantly, this hypothesis could be directly tested by measuring PrP levels before and after protein denaturation, which may restore epitope accessibility and clarify whether the reduced ELISA signal in D178N cases is driven by conformational masking rather than by decreased protein abundance. This approach was not feasible in the present study and should be addressed in future investigations. However, one study applied targeted mass spectrometry (multiple reaction monitoring, MRM) to quantify PrP in CSF and likewise reported reduced PrP levels in prion disease. Notably, this method does not depend on antibody epitopes, as it quantifies peptides generated after protein digestion, corroborating our findings.^47^

This question has been systematically addressed by Mortberg *et al*. by evaluating four commercially available antibodies in paired combinations to determine their sensitivity and cross-reactivity.^46^ In this study, an ELISA assay employing EP1802Y as the capture antibody and 8H4 as the detection antibody has been used. The epitopes recognized by these antibodies are located in the C-terminal region of the target protein, specifically at residues 218–227 for EP1802Y and 182–196 for 8H4. This protocol yielded results consistent with those obtained from the BetaPrion ELISA, substantiating that CSF PrP concentrations are significantly reduced in individuals carrying the D178N mutation compared to non-mutation carriers. Similarly, decreased plasma PrP concentrations in sCJD patients using the same Betaprion kit closely mirrors the reductions seen in D178N symptomatic patients and D178N asymptomatic carriers. This evidence argues against the idea of mutation-specific reduced ELISA reactivity and instead suggests that other biological mechanisms underlie the reduced PrP levels.^47^

Assuming that the low PrP concentrations observed in D178N carriers do not simply reflect reduced ELISA reactivity, the underlying mechanism driving lower PrP levels in this mutation remains incompletely understood. One possible explanation is that the molecular characteristics of PrP vary according to the specific genetic mutation, and the pattern of dysfunction also appears to be PrP-strain specific. Under normal circumstances, cellular prion protein (PrP^C^) is synthesized in the endoplasmic reticulum (ER), modified in the Golgi complex and trafficked to the cell surface, where it carries out its normal functions.^43,50^ There is broad consensus that wild-type and mutant PrP undergo distinct intracellular metabolism, with defective quality control leading to misfolded mutant PrP accumulation. Quality control mechanisms can become overwhelmed at the level of secretion, either in the ER^51,52^ or Golgi complex,^53^ resulting in inappropriate secretion of impaired, immature or misfolded proteins.^52^ Additionally, degradation pathways, including the proteasome and lysosomes, are also impaired. This collectively prevents mutant PrP from reaching the cell membrane. The D178N mutation induces conformational changes that destabilize PrP, resulting in only about 30% of the mutant protein reaching the cell membrane.^43^ Transgenic mice expressing the mouse homolog of the CJD178 mutation develop abnormal PrP accumulation in the brain together with prion-like neuropathological changes.^54^ In cells expressing the D178N mutation, unglycosylated and intermediate glycosylated forms of PrP are underrepresented, as they inefficiently traffic to the cell surface and exhibit increased aggregation propensity.^43^ Furthermore, PrP substrate from FFI has shown spontaneous self-aggregating properties in RT-QuIC reaction without prior seeding.^55^ This early aggregation may further contribute to reduced reactivity with ELISA antibodies, as these aggregated or misfolded forms are less accessible for detection.

Additional factors that may influence PrP levels in peripheral blood include the abundance of circulating blood cells. Among these, the highest PrP concentrations have been detected in CD4 + CD45RA-CD62L- effector memory T helper cells, while platelets, although expressing low levels of PrP^C^ on their surface, account for the majority of circulating PrP^C^ due to their high numbers. Importantly, studies have found no significant differences in the composition or PrP^C^ expression of white blood cells between controls and patients with sCJD. These observations suggest that it could be worthwhile to explore whether the reduction in plasma PrP levels is related to cell numbers or other regulatory mechanisms influencing PrP^C^ distribution in blood in future studies.^56^

Intriguingly, PrP exhibits an inverse pattern compared to other brain-derived proteins in prion disease. Whereas concentrations of tau, enolase, S100B, neurofilament light chain and 14-3-3 increase in both plasma and CSF, our findings demonstrate the opposite trend for PrP.^12,57-60^

Preanalytical conditions remain a significant limitation. Previous work shows that repeated freeze–thaw cycles and multiple tube transfers reduce PrP levels, notably after three freeze–thaw cycles and two tube transfers. Storing samples at room temperature or 4°C also significantly decreases PrP concentrations.^17^ Additionally, PrP exhibits strong adhesion to plastic surfaces of storage tubes, an effect that can be minimized by addition of detergent or using specific collection tubes.^61^ Standardization of future sample collection protocols is recommended to enhance the reliability and reproducibility of PrP quantification. Discrepancies in PrP concentrations in plasma samples from E200K symptomatic patients collected at SZU and UMG are possibly due to differences in sample handling. This highlights pre-analytical conditions as a significant source of variability and underscores the need to minimize such variability.

Another aspect that could strengthen future research is the inclusion of longitudinal data. Given that PrP may be relevant for monitoring disease progression and evaluating the efficacy of potential therapeutic interventions, such data would provide valuable additional insights.

This study quantifies PrP concentrations in biofluids from individuals at risk for, or with established, prion disease. Taken together, our findings demonstrate low plasma PrP levels in patients with genetic and sporadic prion diseases. In addition, our data identify a distinct, mutation-specific PrP signature of the D178N mutation, characterized by consistently reduced PrP levels both in pre-symptomatic and symptomatic carriers. This consistent biomarker profile across biological compartments and disease states highlights the potential utility of PrP quantification for early detection, risk stratification and monitoring in individuals with prion disease-associated *PRNP* mutations.

## Supplementary Material

fcag321_Supplementary_Data

## Data Availability

Raw data were generated at the Department of Neurology of the University Medical Center Göttingen (UMG) (Germany). Derived data supporting the findings of this study are available from the corresponding author upon request.
