# Supplementary material for: Prion protein (PrP) profiles in blood and CSF: insights into pre-symptomatic and symptomatic prion disease
Source: Brain Commun. 2026 Aug 22;8(5):fcag321. doi: 10.1093/braincomms/fcag321 (PMC13543685; doi:10.1093/braincomms/fcag321)
Supplement: Supplementary file 1 [file fcag321_supplementary_data.pdf]

Supplementary Material

Supplementary Fig. 1

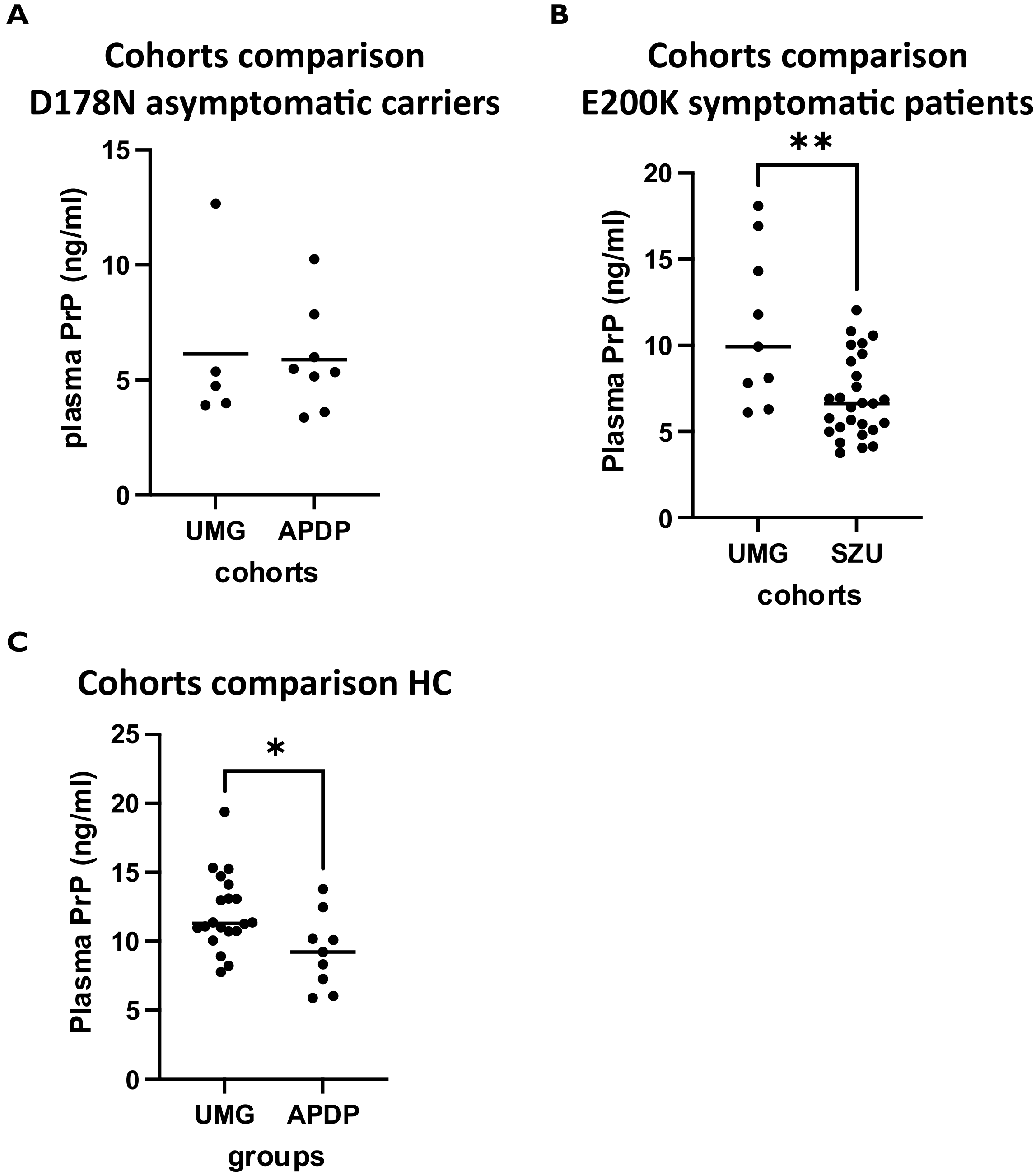

Supplementary Fig. 1: Cohort comparison of Plasma prion protein (PrP) concentrations **A** in D178N asymptomatic mutation carriers from the University Medical Center Göttingen (UMG) cohort and from the Portuguese Association of Prion Diseases (APDP) cohort. **B** in E200K symptomatic patients from the UMG cohort and Slovak Medical University (SZU) cohort. Plasma PrP levels were significantly lower in the SZU cohort compared with UMG cohort ($p = 0.0098$) **C** in healthy controls from the UMG and APDP cohorts. Plasma PrP levels were significantly lower in the APDP cohort compared with UMG cohort ($p = 0.0175$). Mann-Whitney U test was used.

Supplementary Fig. 2

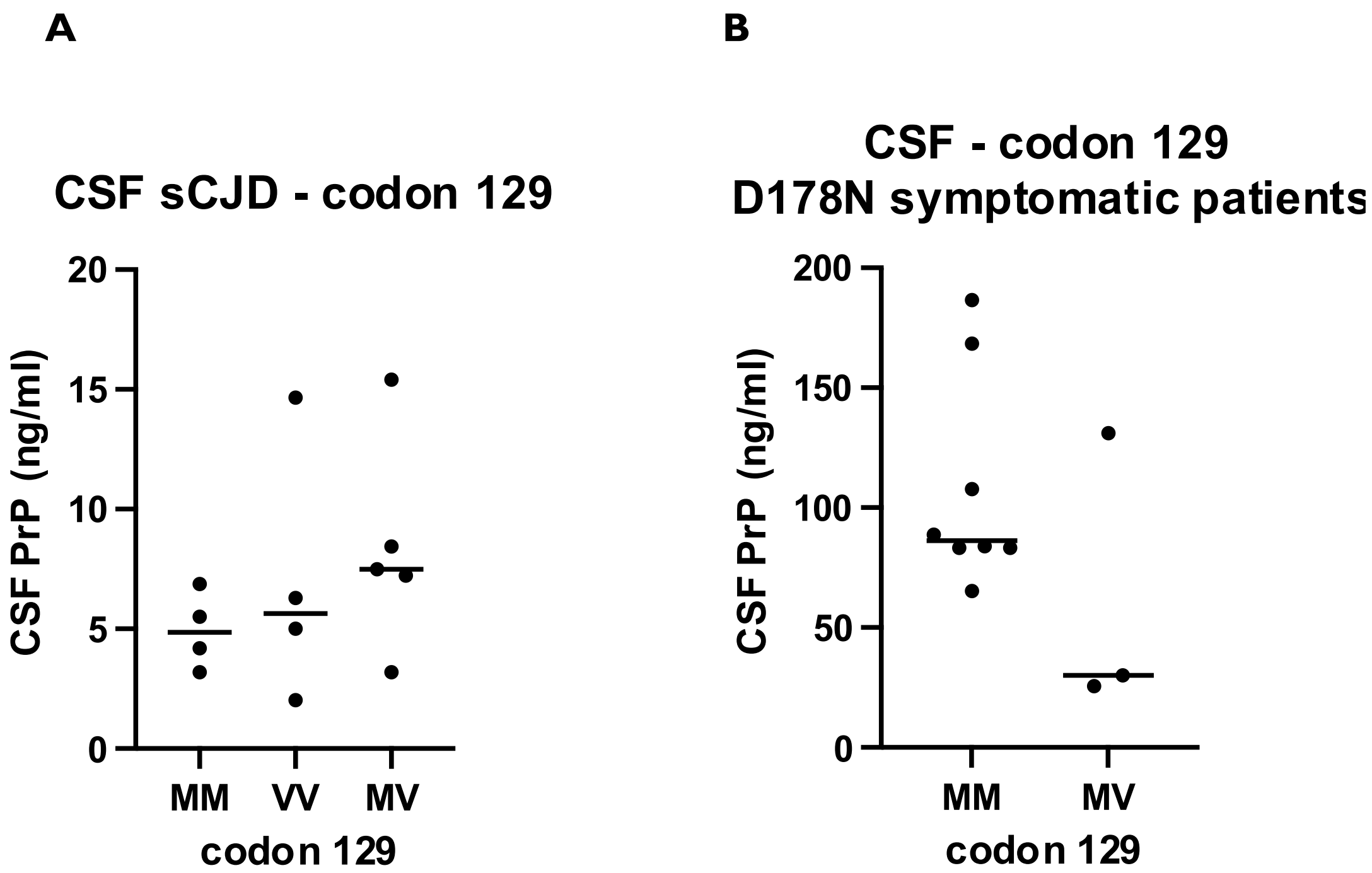

Supplementary Fig. 2: CSF prion protein (PrP) concentrations stratified by prion protein gene (PRNP) codon 129 polymorphism (M methionine, V valine) in **A** No significant differences were observed regarding CSF PrP levels in different codon 129 genotypes among sporadic Creutzfeldt-Jakob Disease (sCJD) cases (MM n = 4, VV n = 4, MV n = 5; p = 0.26) and **B** No significant differences were observed regarding CSF PrP levels in different codon 129 genotypes among D178N symptomatic patients (MM n = 8, MV n = 3; p = 0.26). Kruskal-Wallis test followed by Dunn's post hoc test for multiple comparisons and Mann-Whitney U test for two group comparisons.

	Plasma/ repeated samples	CSF/ repeated samples	Source
**D178N symptomatic patients**	10/1	10/0	UMG
**D178N asymptomatic carriers**	5/1	1/0	UMG
	8/0	-	APDP
**P102L symptomatic patients**	2/2	-	UMG
**P102L asymptomatic carriers**	3/0	-	UMG
**E200K symptomatic patients**	9/2	-	UMG
	27/0	-	SZU
**E200K asymptomatic carriers**	2/0	-	SZU
	1/0	-	APDP
**Alzheimer's Disease (AD)**	10/2	11/1	UMG
**Sporadic Crutzfeldt-Jakob (sCJD)**	21/1	20/1	UMG
**Healthy controls (HC)**	9/0	-	APDP
	20/0	-	UMG, Transfusion Medicine
**Non-neurodegenerative controls (ND)**	-	25/0	UMG

Supplementary Table 1: Sample origin, total number of samples and patients with repeated sampling.
